# Severe autoimmune hemolytic anemia following immunotherapy with checkpoint inhibitors in two patients with metastatic melanoma: a case report

**DOI:** 10.3389/fimmu.2024.1342845

**Published:** 2024-03-20

**Authors:** Tanja Fetter, Simon Fietz, Maya Bertlich, Christine Braegelmann, Luka de Vos-Hillebrand, Joerg Wenzel, Annkristin Heine, Jennifer Landsberg, Philipp Jansen

**Affiliations:** ^1^ Center of Dermatooncology and Phlebology, University Hospital Bonn, Bonn, Germany; ^2^ Department of Oncology, Hematology and Rheumatology, University Hospital Bonn, Bonn, Germany

**Keywords:** autoimmune hemolytic anemia, melanoma, immunotherapy, anti-PD-1 antibody, anti-CTLA 4-antibody, immune-related adverse events, myocarditis, case report

## Abstract

**Introduction:**

Over the past decade, immune checkpoint inhibitors such as antibodies against cytotoxicity T-lymphocyte-associated protein 4 (CTLA-4) and programmed cell death protein 1 (PD-1) have become an important armamentarium against a broad spectrum of malignancies. However, these specific inhibitors can cause adverse autoimmune reactions by impairing self-tolerance. Hematologic side effects of immune checkpoint inhibitors, including autoimmune hemolytic anemia (AIHA), are rare but can be life-threatening.

**Case report:**

Herein, we report two patients on immune checkpoint inhibitors for metastatic melanoma who developed AIHA with symptoms of dyspnea and fatigue. In the first patient, symptoms alleviated after discontinuation of combined anti CTLA-4 and anti-PD-1 therapy, initiation of corticosteroids and application of a single red blood cell transfusion. Due to subsequent progress of melanoma, combinational anti-PD-1 and tyrosine kinase inhibitor therapy was initiated based on multidisciplinary tumor board decision. After two months, she again developed the described hematological and clinical signs of AIHA leading to cessation of anti-PD-1 therapy and initiation of corticosteroids, which again resulted in an alleviation of her symptoms. Due to further progression, the patient received dacarbazine for several months before she decided to stop any therapy other than palliative supportive care. In the second patient, discontinuation of anti-PD-1 therapy and initiation of corticosteroids entailed a complete alleviation of his symptoms. After refusing chemotherapy due to subsequent melanoma progression, he received radiotherapy of bone metastases and is currently enrolled in a clinical trial. The patient did not develop AIHA ever since.

**Conclusion:**

Hematologic immune-related adverse events due to treatment with immune checkpoint inhibitors are rare but can have life-threatening consequences. If dyspnea and other clinical symptoms are present, AIHA should be considered as a potential cause and treated promptly in a multidisciplinary setting. An expanded comprehension of risk factors and pathogenesis of AIHA is needed to identify high-risk patients beforehand, leading to more effective predictive and reactive treatment approaches.

## 1 Introduction

Immune checkpoint inhibitors (ICPi) such as antibodies against programmed cell death protein 1 (PD-1) and cytotoxicity T-lymphocyte-associated protein 4 (CTLA-4) have become indispensable for the treatment of various malignancies such as metastatic melanoma (1, 2). Since the first agents were approved for the treatment of advanced melanoma, they have significantly improved the prognosis of the disease (1). However, due to the ICPi-induced upregulated antitumor immune response, mediated by non-antigen-specific T cells, different immune-related adverse events (irAE) may occur, e.g. dermatitis, colitis, hepatitis, endocrinopathies and, less commonly, hematologic adverse events autoimmune hemolytic anemia (AIHA), immune thrombocytopenia and agranulocytosis (3, 4). Most irAE are treated with high-dose corticosteroids, interruption of ICPi therapy and supportive care.

This report describes two patients with metastatic melanoma who developed AIHA while undergoing treatment with ICPi (**Table 1**). Both patients experienced dyspnea and fatigue. The first patient, who was in stage IV according to the American Joint Committee on Cancer (AJCC) staging system, had received both ipilimumab (targets CTLA-4) and nivolumab (targeting PD-1) combination therapy as well as nivolumab monotherapy before developing the first episode of AIHA. After resuming ICPi treatment with pembrolizumab (targeting PD-1), she developed a second episode of AIHA. The other patient received pembrolizumab treatment for his AJCC stage IIIB melanoma prior to the onset of AIHA.

**Table 1:** Synopsis of demographics, background, treatment and outcome of described cases.

	Case 1	Case 2
**Age and gender**	64F	75M
**ECOG**	0	1 (07/23)
**Disease specification**	Last disease status (07/2023) Metastatic melanoma with inguinal, iliac and para-aortic lymph node (12/2020) as well as hepatic (09/2022) metastases (pT4b N3c M1a(1) - stage IV AJCC 2017) Disease status at time of AIHA (02/2022 and 09/2022) Metastatic melanoma with inguinal, iliac and para-aortic lymph node (12/2020) metastases (pT4b N3c M1a(1) - stage IV AJCC 2017)	Current disease status (09/2023) Metastatic melanoma with hepatic (ED 12/2022), pulmonary (12/2022) and bone (03/2023) metastases (pT3a cN1c cM1c (1) - stage IV AJCC 2017) Disease status at time of AIHA (09/2022) Metastatic melanoma of the right lower back with a subcutaneous metastasis of the right lower back (06/2021) (pT3a cN1c cM0 - stage IIIB AJCC 2017)
**Genomic alterations**	BRAF wild type, no evidence of any pathogenic gene sequence variants	BRAF wild type, NRAS: Q61R, ARID1A: Q420H, SF3B1: R549H, PD-L1 positive (5%)
**Comorbidities**	• Bronchial asthma (since 01/2021)	• Melanoma *in situ* of the right cheek (03/2019), total excision therapy • Cardiac pacemaker (with a disconnected right ventricular probe since 2003) • Arterial hypertension • Hyperuricemia • Transient ischemic attack in 2019 • Partial bowel resection due to intestinal stenosis in 2005
**Melanoma-specific therapy**	12/2020 Diagnosis of vulvar melanoma metastasis with para-aortic and pelvic lymph node metastases 01 – 03/2021 4 cycles of immunotherapy with ipilimumab (3mg/kg) and nivolumab (1mg/kg) every three weeks 04 – 11/2021 8 cycles nivolumab flat dose 480 mg every four weeks 12/2021: progression of lymph node metastases 12/2021 – 02/2022 3 cycles of immunotherapy with ipilimumab (3mg/kg) and nivolumab (1mg/kg) every three weeks 02-03/2022 First episode of AIHA (CTCAE grade 3) 04-05/2022 Radiation therapy of inguinal lymph node metastases (cumulative dose of 39 GY) 06-09/2022 3 cycles of immunotherapy with pembrolizumab (200 mg per infusion) every three weeks in combination with Lenvatinib 20 mg per day 09/2022 Second episode of AIHA (CTCAE grade 3) 11/2022 – 12/2022 Radiation therapy of para-aortic lymph node metastasis (cumulative dose of 36 GY) 01-05/2023 5 cycles of chemotherapy with dacarbazine 06-07/2023 Palliative treatment, death in 07/2023	11/2018 Diagnosis of nodular melanoma of the lower right back, excision and sentinel lymph node biopsy (0/1) in 12/2018 05/2022 Resection of a suspected in transit metastasis of the lower right back 08/2022 Radiation therapy in the region of the lower right back (cumulative dose of 50 GY) 08/2022 1 cycle of immunotherapy with pembrolizumab (400 mg per infusion; planned therapy all six weeks) 09/2022 Episode of AIHA (CTCAE grade 3) 03/2023 Radiofrequency ablation of liver metastases, that had been discovered three months earlier 04-05/2023 Pelvic radiation therapy for newly diagnosed iliac bone metastases (cumulative dose of 39 GY) Since 05/2023 Screening for and inclusion in a clinical trial
**Time to AIHA from first dose/from last dose of ICPi**	First episode of AIHA: 13 months/2 weeks (ipilimumab/nivolumab) Second episode of AIHA: 2 months/4 weeks (pembrolizumab)	4 weeks
**Laboratory/DAT**	02/2022 DAT positive, with anti-IgG (128+) and anti-C3d positive antibodies (2+). 03/2022 DAT positive, with anti-IgG (16+) antibodies, negative for anti-C3d antibodies 08/2022 DAT positive, with anti-IgG (8+) and anti-C3d positive antibodies (16+)	09/2022 DAT negative
**Treatment of AIHA**	First episode of AIHA: Oral Prednisolone (80mg, tapered over weeks) One red blood cell transfusion Second episode of AIHA: Intravenous Prednisolone (70mg, oralized after a few days and tapered over weeks) Two red blood cell transfusions	Intravenous prednisolone (90 mg, oralized after 10 days and tapered over weeks)
**Concurrent irAEs**	09/2022 Thrombocytopenia (CTCAE grade 1)	Myocarditis (CTCAE grade 2)
**Outcome/Time to recovery**	First episode: Resolution of AIHA/4 weeks to comparable previous hemoglobin levels Second episode: Resolution of AIHA/6 weeks to comparable previous hemoglobin levels	Resolution of AIHA/26 weeks to comparable previous hemoglobin level

These cases highlight the importance of accurately identifying and diagnosing rare hematologic irAEs, such as AIHA. They also emphasize the need for a deeper understanding of AIHA risk factors and pathogenesis to identify patients who may be at higher risk of developing AIHA. This understanding may also help identifying which patients are eligible for reinitiating ICPi despite previous AIHA.

## 2 Case descriptions

### 2.1 Case 1

A female patient in her sixties presented to our department in September 2022 with an acute deterioration of her condition, suffering from shortness of breath and fatigue.

**Figure 1:**
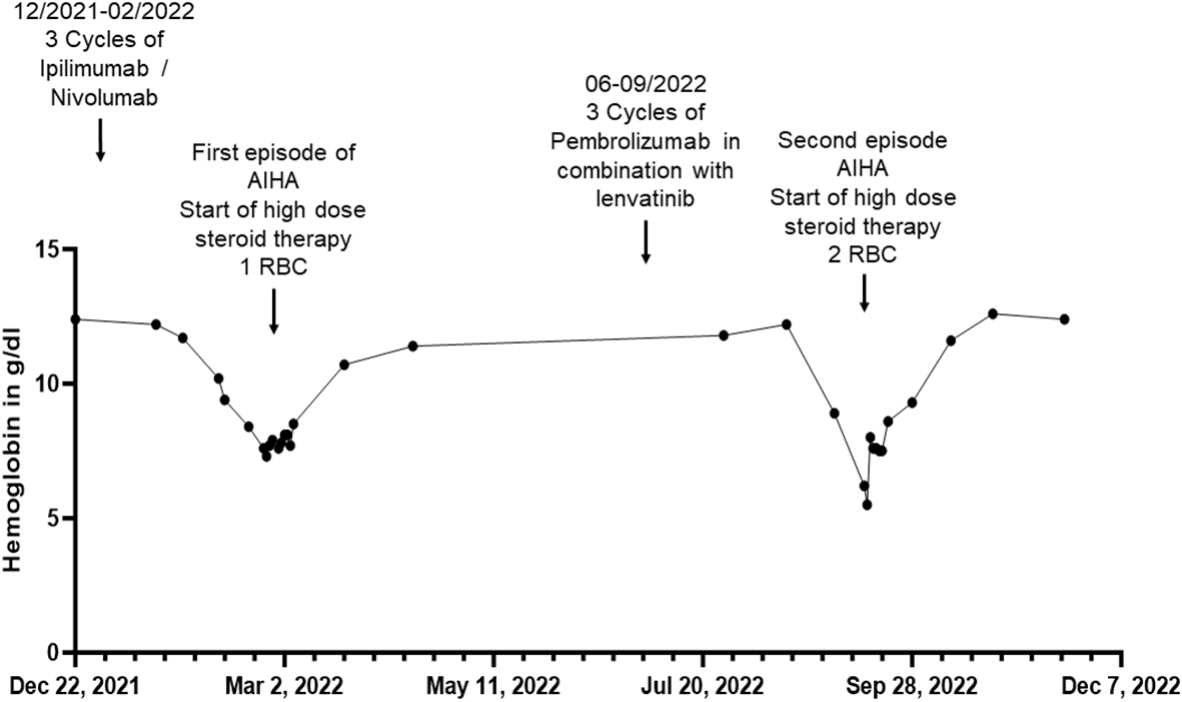
Overview of hemoglobin in g/dL of patient 1 before, during and after the inpatient stays for autoimmune hemolytic anemia (AIHA) (in February/March and September 2022) over a total period of one year. RBC, red blood cell concentrate.

In December 2020, the patient was diagnosed with stage IV AJCC 2017 melanoma metastasis of the vulva, along with advanced para-aortic, iliac, and inguinal lymph node metastases. According to the advice of the interdisciplinary tumor board, she underwent excision of melanoma metastasis, which did not show a proto-oncogene B-Raf (BRAF) mutation (BRAF-wildtype). Subsequently, we initiated a systemic treatment with the ICPi ipilimumab, which is directed against CTLA-4, and nivolumab, which is directed against PD-1. From January to November 2021, the patient received a total of four standard doses of immunotherapy (1mg/kg nivolumab and 3mg/kg ipilimumab based on a 70 kg weight) every three weeks and a total of eight doses nivolumab (480mg per infusion) as monotherapy every four weeks hereafter. Due to progression of disease in December 2021 systemic treatment with ipilimumab (3 mg/kg) and nivolumab (1mg/kg) every three weeks was re-initiated (**Figure 1**).

At the end of February 2022 - two weeks after the third dose of combined immunotherapy and thirteen months after the first infusion of immunotherapy - the patient turned to our department with acute onset of dyspnea and fatigue for the first time. Laboratory results revealed normocytic normochromic anemia with a hemoglobin of 7.6 g/dL decreased from 11.7 g/dL four weeks earlier and an elevated Lactate dehydrogenase (LDH) of 326 U/l. Acute bleeding or other potential causes (e.g. asthma condition) as a cause of her symptoms could be excluded. The direct antiglobulin test (DAT) was found positive, with anti-IgG (128+) and anti-C3d positive antibodies (2+). These findings were interpreted as AIHA of warm-antibody type (Common terminology criteria for adverse events (CTCAE) grade 3). However, due to partially normal hemolysis parameters (normal haptoglobin and bilirubin levels), an already expired hemolysis event at the time of hospitalization was suspected. The patient received a transfusion of a red blood cell concentrate and systemic treatment with prednisolone 80 mg per os (p.o.), which was tapered over the following weeks. Treatment resulted in an increase in hemoglobin from 7.3 to 10.7 g/dL within four weeks. A DAT repeated at the end of March 2022 showed a decrease in anti-IgG (16+) antibodies and was found negative for anti-C3d antibodies. Reticulocyte levels, however, remained low despite the respective treatment (5.500 cells/uL after a week of treatment). Thus, to exclude a myelopoietic maturation disorder, bone marrow aspiration was performed revealing no cytological or molecular genetic evidence of myelodysplastic syndrome or bone marrow carcinosis of the melanoma. In addition, a karyogram showed no pathologic findings. Treatment with the ICPi ipilimumab and nivolumab remained discontinued since February 2022. In April and May 2022, the patient received radiotherapy of the inguinal lymph nodes with a total dose of 39 GY. In July 2022, the patient was switched to immunotherapy with the anti PD-1 antibody pembrolizumab 200 mg per infusion every three weeks in combination with the multikinase inhibitor lenvatinib 20 mg per day in accordance with interdisciplinary tumor board recommendations due to progressive inguinal lymph node metastases. A repeated DAT in August 2022 showed a further decrease in anti-IgG (8+) antibodies, but positive anti-C3d antibodies (16+). In September 2022, two months after initiation of pembrolizumab, of which she had received three cycles, and continuous intake of lenvatinib, she again presented with dyspnea and fatigue, revealing a normocytic, normochrome anemia with a hemoglobin of 5.5 g/dL and thrombocytopenia with thrombocytes of 94.000 cells/uL, decreased from normal values of 12.2 g/dL and 219.000 cells/uL four weeks earlier, respectively. In addition, an elevated LDH of 407 U/l and a decreased absolute reticulocyte count of 10.400 cells/uL could be observed, other hemolysis parameters were found normal. Other causes for the lowered hemoglobin level were again excluded. Given the suspected recurrence of AIHA in combination with thrombocytopenia, the likely diagnosis was Evans syndrome.

We decided to start a systemic, initially intravenous, steroid therapy with 70 mg prednisolone, leading to a significant alleviation of her symptoms. Furthermore, the patient received two red blood cell concentrates, entailing an increase of hemoglobin from 5.5 g/dL to 8.0 g/dL. In the following four weeks after the red blood cell transfusion, the hemoglobin value increased to 11.6 g/dL. Of note, systemic treatment with lenvatinib therapy was continued during the entire period while pembrolizumab was discontinued. Due to metastatic progression with additional manifestation of liver metastases in the following months, dacarbazine was initiated in January 2023, of which she received five doses (850mg/m^2^ per administration). Until May 2023, no hematological adverse events occurred. In June 2023, the patient decided to stop dacarbazine chemotherapy and to receive supportive palliative care only. She passed away in July 2023.

### 2.2 Case 2

A male patient in his seventies presented to our department in September 2022 in a reduced general condition, accompanied by increasing exertional dyspnea for several weeks, occasional chest pain and symptoms of orthostatic dysregulation.

**Figure 2:**
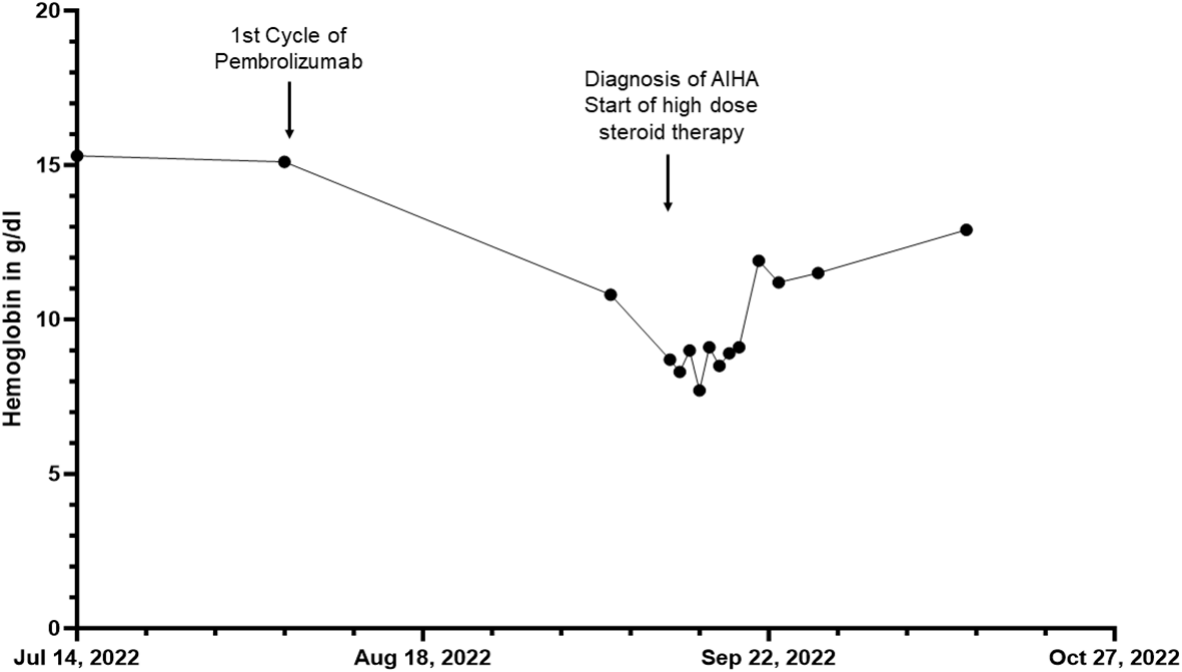
Overview of the hemoglobin in g/dL of patient 2 before, during and after the inpatient stay for autoimmune hemolytic anemia (AIHA) over a total period of three months.

**Figure 3:**
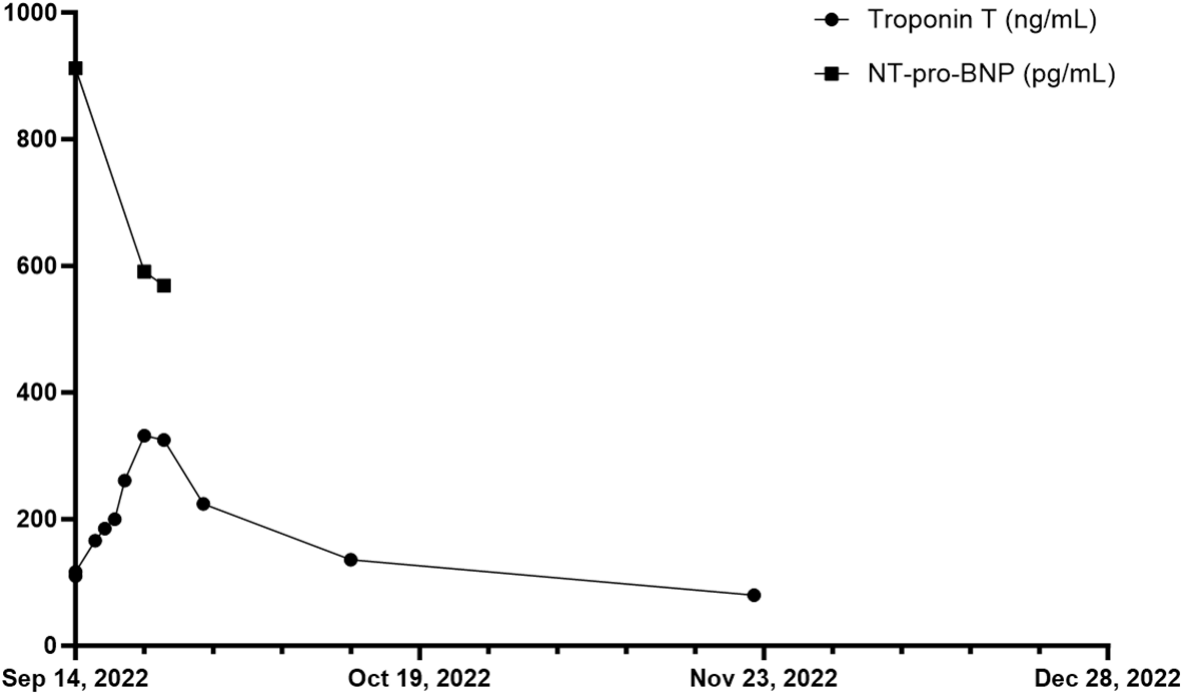
Overview of troponin T in ng/mL and NT-pro-BNP in pg/mL of patient 2 during and after the inpatient stay over a total period of ten weeks. First measurement of troponin T and NT-pro-BNP on 14th of September 2022.

In November 2018, the patient had been diagnosed with melanoma of the lower back (BRAF wildtype, NRAS-Q61R mutation, programmed cell death ligand 1 (PD-L1) positive 5%). The patient was admitted to a hospital for excision of melanoma along with the sentinel lymph node on the right side of the groin, showing no evidence of metastasis (stage IIA AJCC 2017). Initiated interferon-α therapy with Roferon 3 million IU subcutaneous (s.c.) 3x/week was discontinued by the patient after hospitalization. In May 2022, an in-transit metastasis was resected with negative resection margins at the lumbar back. He was referred to our skin tumor center for further treatment. No further metastases were found. Thus we initiated an adjuvant therapy with pembrolizumab 400 mg every six weeks and radiotherapy of lymph node metastases in August 2022 (**Figure 2**). Radiotherapy was performed five times weekly for a total of four weeks with a total dose of 50 GY. The second dose of pembrolizumab was not administered due to his reported symptoms, which occurred four weeks after the first dose of pembrolizumab. Relevant additional pre-existing diseases included a cardiac DDD pacemaker implanted in 2003 due to syncopal episodes. Laboratory results revealed normocytic normochromic anemia with a hemoglobin of 7.7 g/dL at its lowest, which had decreased from 15.1 g/dL within five weeks and from 10.8 g/dL one week before admission. Additionally, haptoglobin could not be found but an elevated free hemoglobin of 203 mg/dL, which increased to 646 mg/dL over the following five days. In addition, LDH was elevated with 925 U/l, compared to 255 U/l measured five weeks earlier and 790 U/l measured one week earlier. Indirect bilirubin was slightly increased at 0.82 mg/dL, with a reference value of 0.8 mg/dL. Of note, a slightly elevated absolute reticulocyte count of 106.600 cells/uL, indicating a regular bone marrow function. However, the DAT was found negative. Because of the initial cardiac symptoms, N-terminal pro B-type natriuretic peptide (NT-proBNP) and troponin T levels were also taken, revealing an elevated NT-pro-BNP of 912 pg/mL and increased troponin T of 110 ng/mL, the latter, however, was found stable at 117.ng/mL after one hour (**Figure 3**). An electrocardiogram (ECG) showed no evidence for acute ischemia. Transthoracic echocardiography showed severely dilated left and right atria with moderate mitral regurgitation and severe tricuspid regurgitation. Myocarditis was already being treated with a beta-blocker and an Angiotensin-converting-enzyme (ACE) inhibitor (lisinopril 20 mg and bisoprolol 2.5 mg once daily). Acute bleeding as a cause of his symptoms was excluded by esophagogastroduodenoscopy and computed tomography (CT) scans of the head, neck, chest and abdomen, of which the CT scans also excluded immune-mediated pneumonitis, pleural effusions or lesions suspicious for malignancy. Bodyplethysmography also showed no evidence of a pulmonary etiology. Other blood tests excluded acute leukemia and non-Hodgkin lymphoma. Testing for Epstein–Barr virus, cytomegalovirus, parvovirus B19, human immunodeficiency viruses as well as hepatitis viruses A, B and C revealed no acute infection.

Based on baseline findings, we suspected ICPi-mediated AIHA (in CTCAE grade 3 (hemoglobin<8g/dL)) and ICPi-mediated myocarditis (in CTCAE grade 2 (troponin up to 323 ng/l)) due to treatment with pembrolizumab. Steroid therapy with 90 mg intravenous prednisolone was initiated leading to an increase in hemoglobin from 7.7 g/dL to 12.9 g/dL within one month. Seven days after admission, haptoglobin was again detectable at the lower limit of 0.3 g/dL, and free hemoglobin was near normal with 61 mg/dL (upper limit at 50 mg/dL). LDH decreased from 925 U/l on admission to 385 U/l four weeks later. NT-proBNP measured on the day of discharge showed a decrease from 912 pg/mL to 569 pg/mL measured during hospitalization. However, during hospitalization, a further ECG revealed new-onset grade I atrioventricular block, and troponin T levels continuously increased to 261 ng/dL eight days after admission. Therefore, coronary angiography was performed to rule out immune-mediated coronary artery disease despite an improvement in symptoms. Cardiac magnetic resonance imaging (MRI) to confirm myocarditis was not possible due to a dislocated second right ventricular lead of his pacemaker, so DOTA-TOC positron emission tomography (PET)-CT was performed. However, no pathological myocardial uptake of the tracer was found, but a metastatic neoplasm in the liver could be detected. In December 2022, this suspicion was confirmed during follow-up staging, which revealed additional liver lesions as well as pulmonary lesions besides a peripheral pulmonary artery embolism in both lungs. In the following tumor board, it was decided not to initiate a new immunotherapy due to immune-mediated cardiomyositis and AIHA after adjuvant pembrolizumab. The suspected liver metastases could be confirmed histologically. The patient decided not to undergo chemotherapy with dacarbazine but opted for local radiofrequency ablation of liver metastases. At staging in March 2023, new metastases were found in the kidney and ilium. Due to severe pain from bone metastases, the patient received radiotherapy from April 2023 until May 2023 with a total dose of 39 GY. He currently participates in a clinical trial receiving chimeric antigen receptor (CAR) T cell therapy (IMA203 clinical trial, NCT03686124).

## 3 Discussion

Autoimmune adverse events induced by ICPi can affect all organ systems and may be fatal, depending on the chosen immunotherapy and general condition of the patient. Although rare, hematological irAEs such as AIHA, thrombocytopenia, neutropenia, and bone marrow failure can be fatal in approximately 15% of cases (4). In general, the onset of ICPi-mediated irAEs varies but is approximately within the first eight to twelve weeks after the initiation of immunotherapy treatment (5, 6). When patients report experiencing shortness of breath during or after ICPi therapy, healthcare providers should consider AIHA as a potential cause, in addition to more common causes such as heart or lung disease. It is important to note that shortness of breath is the typical symptom shared among these patients. To diagnose suspected cases of AIHA, it is essential to evaluate DAT and hemolysis parameters (7). While secondary AIHA associated with ICPi shares several clinical features with primary (idiopathic) AIHA (8), one aspect that distinguishes ICPi-associated AIHA is the higher incidence of DAT negativity (7), as demonstrated in case 2. Knowledge of accurate diagnosis and treatment options is essential given the high mortality in patients with ICPi-associated AIHA, especially in refractory cases. Moreover, AIHA should be treated immediately in an interdisciplinary setting. Glucocorticoids are a beneficial primary treatment for most patients with ICPi-associated AIHA, in tandem with supportive care. It is recommended that steroid therapy is gradually tapered to a low-dose prednisolone equivalent over a period of 4-6 weeks (9). Depending on the severity of symptoms and measures of hemolysis and hemoglobin parameters, red blood cell transfusion may be necessary during treatment. Secondary immunomodulatory agents can be considered as additional treatment for AIHA or as an alternative option in case of therapy resistance. These encompass rituximab, an anti-CD20 antibody, as well as intravenous immunoglobulins, calcineurin inhibitors and mycophenolic acid (9). More than two-thirds of patients experience full resolution after receiving suitable treatment (10, 11), as observed in our patients and evidenced by their improved general condition as well as hemoglobin and hemolysis parameter measurements.

In case 1, ICPi therapy was readministered due to the substantial enhancement in overall survival and lack of systemic treatment alternatives other than chemotherapy. Current oncological guidelines recommend permanent discontinuation of ICPi only for severe irAEs, which are defined by CTCAE grade 4 (12–14). According to the limited numbers of case series (9), the likelihood of experiencing a recurrence of AIHA following re-administration was found to be approximately 50% (10, 15–17). Other irAEs were seen to recur in the same form after re-administration of ICPi in a range from 18% to 42% in cohort studies and case series (18–22). A meta-analysis including more than 12.000 patients receiving ICPi found that restarting ICPi therapy after discontinuation due to irAEs resulted in a survival benefit compared to patients who did not restart therapy (23). Of note, the risk of progression and death for ICPi-treated patients with irAEs was found to be reduced in comparison to ICPi-treated patients without irAEs (24). In addition, it was reported that, compared with initial ICPi treatment, rechallenge showed a similar incidence for grade 3 and grade 4 irAEs (23, 25, 26). However, it should be considered that in irAEs with reported higher fatality rates such as AIHA and immune-mediated myocarditis a rechallenge is questionable given the risk of death in case of recurrent toxicity (12, 27). Furthermore, it is relevant to consider whether rechallenge of ICPi involves switching between anti-CTLA4 and anti-PD-1 therapy or re-administration of the same drug class as discussed in detail elsewhere (12). Reinduction should be considered approximately four to six weeks after initiating AIHA therapy, if a favorable response to tapered glucocorticoids is observed (9). During the rechallenge of ICPis, some authors have reported concomitant prophylactic immunosuppression, such as with systemic steroids. However, further investigation of this approach is needed as the available data is limited and controversial (12). Accordingly, it is necessary to evaluate each case individually to determine whether to restart ICPi therapy with the risk of new episode of irAE or to proceed directly to alternative available therapies such as chemotherapy.

ICPi are monoclonal antibodies that disrupt receptor-ligand interactions that most often promote suppression of the immune response in order to prevent hyperactivation of immune cells and the occurrence of autoimmune diseases. By blocking these interactions, effector T cell functions are restored, which promote anti-tumor responses. ICPi either target suppressor receptors such as CTLA-4 on the surface of T cells as well as PD-1 on the surface of diverse immune cells or inhibitory ligands, such as PD-L1, which is expressed by multiple tissues (28–30). Initially, it was thought that CTLA-4 blockade targeted only T cells that were crucial for the antitumor immune response (31). However, this led to uncontrolled activation of all T cells, which compromised tolerance to healthy autologous tissue. Of note, the origin of autoantibodies, as they occur in AIHA, remains unknown. It is possible that the blockade of PD-1 on B cells may unleash B cells directly, triggering the production of autoantibodies. B cell activation could also be triggered indirectly via stimulation of CD4+ T cells as a result of both PD-1- and CTLA-4 blockade (9). Impaired function of regulatory T cells is also considered a contributing factor to the activation of B cells (9). As a result of uncontrolled T- and B-cell activation, autoimmunity is becoming a challenge for cancer immunotherapy (32). It has been suggested that irAEs may be caused by an iatrogenic autologous graft-versus-host disease response (33). To address this issue, a safer ultra-low dose ICPi protocol was developed and tested on 131 unselected stage IV cancer patients with 23 different histologic cancers who had exhausted all conventional treatments. The protocol maintained efficacy (34) and was also found to be more cost-effective than registered doses (35).

In Europe, the CTLA-4 antibody ipilimumab was approved for the treatment of malignant melanoma in 2012, followed by anti-PD-1 antibodies nivolumab and pembrolizumab in 2015 (36). In 2014, Simeone et al. were the first to describe the occurrence of hematological toxicities, including AIHA, following ipilimumab immunotherapy (37). In 2016, Kong et al. first reported AIHA in a patient with metastatic melanoma after receiving anti-PD-1 treatment (38), as likewise occurred in our two patients. Since then, additional cases have been documented - the largest case series currently published involves 68 patients (11).This is in part due to the increasing inclusion of ICPis in treatment protocols as first-, second-, or third-line agents for a diverse array of metastatic cancers. However, hematologic irAEs remain uncommon, and there is a shortage of reports to thoroughly determine the clinical manifestation and treatment algorithms, potential factors, and pathobiological mechanisms of hematologic irAEs. This is also reflected by the present guideline, which is based on professional opinion rather than evidence-based information (14, 39).

To our knowledge, this report is one of the few reports describing the recurrence of AIHA after ICPi rechallenge. However, it is limited by the fact that it only covers two patients and thus lacks sufficient evidence. There is an urgent need for clinical trials and meta-analyses of relevant studies addressing crucial questions such as criteria for identifying the patients who benefit from ICPi rechallenge after experiencing an irAE taking their specific conditions into account.

In summary, these cases highlight the importance of precise identification and diagnosis of uncommon hematologic irAEs such as AIHA. These cases also demonstrate the need for a more profound understanding of AIHA risk factors and pathogenesis to identify patients who are particularly susceptible for developing AIHA. This will enable more effective predictive maintenance alongside reactive management.

## 4 Patient perspective

Prior to initiation of immunosuppressive corticosteroid therapy, both patients were in a poor general condition, with shortness of breath and marked physical weakness as the predominant signs of anemia. In the acute situation, the patients’ main concern was a rapid improvement in their symptoms, which was achieved by the immediate initiation of high-dose steroid therapy and, in patient 1, red blood cell transfusions in the first week of hospitalization.

In the following, both patients expressed uncertainty about the further treatment for melanoma, specifically regarding the possible reintroduction of immunotherapy. The optimal procedure for both patients was discussed in a multidisciplinary setting and decided together with the patients.

## Data availability statement

The original contributions presented in the study are included in the article/supplementary material. Further inquiries can be directed to the corresponding author.

## Ethics statement

Written informed consent was obtained from the individual(s) for the publication of any potentially identifiable images or data included in this article.

## Author contributions

TF: Conceptualization, Writing – original draft, Writing – review & editing, Data curation, Formal analysis, Investigation, Project administration, Software, Validation, Visualization. SF: Data curation, Investigation, Writing – review & editing. MB: Data curation, Investigation, Writing – review & editing. CB: Data curation, Investigation, Writing – review & editing. LV-H: Resources, Writing – review & editing. JW: Resources, Writing – review & editing. AH: Resources, Writing – review & editing. JL: Resources, Writing – review & editing. PJ: Conceptualization, Data curation, Formal analysis, Investigation, Project administration, Resources, Supervision, Validation, Writing – review & editing.
